# Long COVID as a functional somatic symptom disorder caused by abnormally precise prior expectations during Bayesian perceptual processing: A new hypothesis and implications for pandemic response

**DOI:** 10.1177/20503121231194400

**Published:** 2023-08-24

**Authors:** Ari R Joffe, April Elliott

**Affiliations:** 1 Department of Pediatrics, University of Alberta, Edmonton, Alberta, Canada; 2 Department of Pediatrics, University of Calgary, Calgary, Alberta, Canada

**Keywords:** Bayesian predictive processing, functional syndrome, Long-COVID, mass sociogenic illness, somatic symptom disorder

## Abstract

This review proposes a model of Long-COVID where the constellation of symptoms are in fact genuinely experienced persistent physical symptoms that are usually functional in nature and therefore potentially reversible, that is, Long-COVID is a somatic symptom disorder. First, we describe what is currently known about Long-COVID in children and adults. Second, we examine reported “Long-Pandemic” effects that create a risk for similar somatic symptoms to develop in non-COVID-19 patients. Third, we describe what was known about somatization and somatic symptom disorder before the COVID-19 pandemic, and suggest that by analogy, Long-COVID may best be conceptualized as one of these disorders, with similar symptoms and predisposing, precipitating, and perpetuating factors. Fourth, we review the phenomenon of mass sociogenic (functional) illness, and the concept of nocebo effects, and suggest that by analogy, Long-COVID is compatible with these descriptions. Fifth, we describe the current theoretical model of the mechanism underlying functional disorders, the Bayesian predictive coding model for perception. This model accounts for moderators that can make symptom inferences functionally inaccurate and therefore can explain how to understand common predisposing, precipitating, and perpetuating factors. Finally, we discuss the implications of this framework for improved public health messaging during a pandemic, with recommendations for the management of Long-COVID symptoms in healthcare systems. We argue that the current public health approach has induced fear of Long-COVID in the population, including from constant messaging about disabling symptoms of Long-COVID and theorizing irreversible tissue damage as the cause of Long-COVID. This has created a self-fulfilling prophecy by inducing the very predisposing, precipitating, and perpetuating factors for the syndrome. Finally, we introduce the term “Pandemic-Response Syndrome” to describe what previously was labeled Long-COVID. This alternative perspective aims to stimulate research and serve as a lesson learned to avoid a repeat performance in the future.

## Introduction

According to the World Health Organization,

Post COVID-19 condition [i.e., Long-COVID] occurs in individuals with a history of probable or confirmed SARS-CoV-2 infection, usually 3 months from the onset of COVID-19 with symptoms and that last for at least 2 months and cannot be explained by an alternative diagnosis. Common symptoms include fatigue, shortness of breath, cognitive dysfunction but also others and generally have an impact on everyday functioning. Symptoms may be new onset following initial recovery from an acute COVID-19 episode or persist from the initial illness. Symptoms may also fluctuate or relapse over time.^1^

Expert groups have emphasized that Long-COVID is a feared and common complication in the population. For example, a Canadian policy report wrote in their executive summary that “an important element of this pandemic response should now be focused on preventing and treating post-COVID-19 syndrome in patients . . . .”^2^ A multinational Delphi consensus recommended that “REC2.9 Public health policy should take better account of the potential long-term impact of the unchecked spread of COVID-19, given ongoing uncertainties about the prevalence, severity, and duration of post-COVID-19 morbidity (long COVID),” and to “REC5.6 Prioritize research funding for long COVID . . .,” also writing that “continued uncertainty about the widespread consequences of long COVID and its implications for public health policy is an ongoing concern.”^3^ Many studies and reviews have hypothesized that symptoms of Long-COVID are due to irreversible tissue damage, possibly from the viral infection itself, the immune response to the virus with systemic hyperinflammation and neuroinflammation, hypercoagulation, and/or tissue scarring.^4–10^

This review addresses the problem that most cases of Long-COVID remain “medically unexplained,” without reproducible structural organ pathology, which has led patients to accuse physicians of “medical gaslighting”—patient concerns have been dismissed and patients have been told there is “nothing we can do.”^11^ To address this problem, we aim to develop the idea that the constellation of symptoms ascribed to Long-COVID are most often genuinely experienced persistent physical symptoms that are functional in nature and hence manageable and potentially reversible, that is, that Long-COVID is a somatic symptom disorder (SSD). First, we describe what is known about Long-COVID in children and adults, including a close look at limitations of existing studies, the incidence of and risk factors for the syndrome, and evidence that the symptoms are “medically unexplained.” Second, we examine so-called Long-Pandemic effects that create a risk for similar symptoms to develop in non-COVID-19 patients. Third, we describe functional syndromes (identified before the COVID-19 pandemic), including SSD, related disorders (syndromes), and functional neurological disorders (FNDs), and suggest that by analogy, most Long-COVID phenotypes are best conceptualized as one of these functional syndromes, with similar symptoms and predisposing, precipitating, and perpetuating factors. Fourth, we describe mass sociogenic illnesses in the past and discuss the concept of nocebo effects. Again, we suggest that, by analogy, Long-COVID is compatible with these descriptions. Fifth, we describe the current theory of the mechanism for functional disorders, the Bayesian predictive coding model (BPCM) for perception, including moderators that can make perceptual inferences functionally inaccurate. We describe how functional symptoms, and predisposing, precipitating, and perpetuating factors can be understood within this model. Finally, we discuss some implications of this framework for improved public health messaging and management of Long-COVID. We believe the current approach inducing fear of Long-COVID in the population, with constant messaging about disabling symptoms of Long-COVID and theorizing irreversible tissue damage as the cause of Long-COVID, has created a self-fulfilling prophecy by introducing the very predisposing, precipitating, and perpetuating factors for the syndrome.

Table 1 gives a summary of the main points made in each section, to help readers follow the narrative review leading to our conclusions. We offer this alternative perspective to argue that the pandemic response created the perfect storm of factors causing the fallout known as Long-COVID. We hope that the lessons learned will prevent a repeat performance in the future.

**Table 1.** Summary of our main findings.

Section of paper	Main points
What is currently known about Long-COVID	1. Compared to controls, the incidence of Long-COVID is very low in children and also low in adults. 2. Notable risk factors included female gender, worse pre-COVID mental health, and more severe acute COVID (often including dyspnea). 3. Although tissue damage has been hypothesized, the symptoms are more likely not associated with structural tissue pathology (i.e., symptoms are in the so-called “medically unexplained” category). 4. Significant limitations of studies do not allow for more definitive statements. 5. A low incidence of persistent symptoms compared to controls neither imply low clinical significance nor mean that children and adults are fine; rather, it means that many have a Long-Pandemic syndrome that may be only somewhat worsened by having had COVID-19.
Long-pandemic effects	1. The response to the COVID-19 pandemic has had adverse effects on mental health, physical activity, body mass index, neuro-inflammation, and fear in the population. 2. In turn, these are risk factors for Long-COVID and Long-Pandemic symptoms.
Functional syndromes	1. There is a marked similarity in symptoms, risk factors (including predisposing, precipitating, and perpetuating factors), and syndromes between functional disorders and Long-COVID. 2. Symptoms in both functional syndromes and Long-COVID cannot be explained by structural tissue pathology (i.e., are in the so-called “medically unexplained” category). 3. An argument from analogy suggests that most Long-COVID is a phenotype of SSD.
Mass sociogenic illness	1. Mass sociogenic illnesses are functional syndromes associated with the contagion of fear that seem to occur in the context of negative information flooding media and social networks. 2. Nocebo effects are likely a common contributing factor in mass sociogenic illness. 3. Long-COVID symptom reports are compatible with this description, and nocebo effects are likely actively contributing to persistent symptoms.
Mechanisms of functional disorders	1. The BPCM of brain perception can explain the functional disorders and syndromes discussed in this paper. 2. In addition, the model can account for the various predisposing, precipitating, and perpetuating factors identified for the functional disorders and syndromes discussed. 3. This unifying theoretical account makes the case stronger that Long-COVID and so-called Long-Pandemic are functional syndromes that could have been expected given the common public health and media responses to the pandemic.
Implications for management of Long-COVID	1. Reducing the predisposing factors of health anxiety, fear, depression, negative media coverage, social isolation, and physical inactivity are important. a. Repeatedly provide accurate information about risk in context from trusted authorities. b. Abandon lockdowns, including abandoning school closures, as they were ineffective and caused massive collateral damage that contributed to the predisposing, precipitating, and perpetuating factors for Long-COVID. c. Improve media coverage with accurate information about risk and trade-offs. d. Ensure surge capacity in healthcare (without closing all healthcare other than for COVID-19). e. Abandon universal masking in the community and schools, as they were ineffective, and signal and reinforce fear. 2. De-escalate social reinforcement (contagion) perpetuating Long-COVID, by providing a clear explanation about functional disorders and their potential reversibility. 3. Provide multidisciplinary treatment for SSDs, including acknowledging the genuinely experienced symptoms and disability, and providing physical therapy/rehabilitation, cognitive behavioral therapy, follow-up, and reassurance.
Limitations	1. This was not a systematic review, although we refer to many systematic reviews throughout. 2. There may be other pathophysiological mechanisms for some cases of Long-COVID. 3. The hypothesis that Long-COVID is usually a functional disorder requires testing, for example, by further documentation of positive features of functional disorders (e.g., inconsistency over time, distractibility), and prevention and treatment trials using interventions we have suggested.

BPCM, Bayesian predictive coding model; SSD, somatic symptoms disorder. For more details and many references, please see the text.

## What is known about Long-COVID

### In children

A striking finding is that severe limitations in the available studies make definitive statements difficult. Systematic reviews list study limitations that include the following: lack of a control group while including a large range and number of nonspecific symptoms that are highly prevalent in the general population, and that could be new or intermittent or fluctuating, without pathognomonic features (i.e., almost anything could be Long-COVID); missing data on symptom severity; selection bias due to often self-selected unblinded patients with high nonresponse bias or more severe cases being more closely tested and monitored; outcome measurement bias due to unblinded subjective self-report of symptoms; missing data bias due to attrition; unmeasured confounding bias; recall bias in the face of substantial public awareness; and inaccurate denominators due to including cases rather than all infections.^11–17^ Hirt et al. concluded that “none [of the studies] provided evidence with reasonable certainty . . . inadequate, over-confident interpretation of the findings by media and decision-makers may cause potentially unnecessary fears and worries among parents and children with SARS-CoV-2 infection.”^12^ Lopez-Leon concluded that “all studies had a high probability of bias.”^15^ This may explain why there was marked heterogeneity between studies.^13–17^ In addition, most studies determined the cross-sectional prevalence of symptoms, while longitudinal study showed that most individual cases resolved over time, and many new cases developed adverse symptoms months after the infection at rates similar to controls.^18^

Several studies with controls have found that Long-COVID may not occur at a higher rate among COVID-19 cases compared to control groups,^19–24^ or that Long-COVID was rare, with some symptoms worse in the control group (including worse objective scoring of somatic symptoms distress).^22,25–30^ Studies with controls have also found a *higher* sense of well-being^24^ or quality of life (with reduced psychological symptoms),^26,27^ or no difference in well-being or quality of life in Long-COVID cases.^31^ Some studies without controls suggested a high incidence of Long-COVID, but correcting those study denominators for MIS-C incidence (which occurs in <1/3000 infections),^32^ the rates were <0.5%.^33,34^ Systematic reviews have reached similar conclusions. Zimmerman et al.^14^ found a difference in persistent symptoms of <4% compared to controls and suggested that “infection-associated symptoms are not necessarily more common or severe than pandemic-associated symptoms,”^13^ and that “nearly all symptoms are also reported in similar frequencies in those without evidence of infection [i.e., may be due to pandemic-related symptoms].” The Global Burden of Disease Collaborators found a Long-COVID incidence of 2.7% in non-hospitalized COVID-19 cases aged <20 years.^7^ Hirt et al.^12^ found that the “two largest studies had symptoms without infection in 34% and 53% [much higher than any estimate for children with infection in the uncontrolled studies].” Stephenson et al.^16^ and Behnood et al.^17^ found well-being to be similar in cases and controls. For individual symptoms, systematic reviews had conflicting findings, with Lopez-Leon et al.^15^ finding no differences between cases and controls in mood symptoms, fatigue, headache, rhinitis, concentration, myalgia/arthralgia, cough, sore throat, or nausea/vomiting, but more dyspnea, fever, and anosmia/ageusia, while Behnood et al.^17^ found no difference in abdominal pain, cough, fatigue, myalgia, insomnia, diarrhea, fever, dizziness, or dyspnea, but more cognitive difficulties, headache, loss of smell, sore throat, and sore eyes. Notably, one population-based study found no long-term increased use of primary or specialist care among COVID-19 cases compared to controls.^35^

Among COVID-19 cases, risk factors for Long-COVID in individual studies have included female gender (in teenagers),^26,27,31,36^ poor or very poor pretest physical and mental health,^31^ any long-term condition,^29,36^ hospitalization,^22^ intensive care unit admission,^29^ and more acute COVID-19 symptoms.^22^ Systematic reviews found that risk factors included female gender,^15–17^ older age,^15–17^ worse self-rated physical and mental health,^15–17^ feeling of loneliness pre-infection,^16^ severe or more symptoms during acute COVID-19,^15,16^ and lower study quality.^17^

Although tissue damage was hypothesized as a cause of Long-COVID,^10^ some authors admitted that this may not be the case,^10,15,16,37–39^ as summarized in Table 2.

**Table 2.** Studies and editorials report that Long-COVID symptoms are not associated with structural pathology (i.e., are “medically unexplained”).

Type of study	Findings	Reference
Children with Long-COVID		
Narrative review of Long-COVID	Long-COVID may be a syndrome “as in many post-viral syndromes in children.”	Stephenson et al.^16^
Narrative review of Long-COVID	“Laboratory or biochemical abnormalities do not correlate with symptoms.”	Robinson and Le Saux^10^
Bioethics review of vaccination	“[It is] biologically implausible that an infection that is usually mild or asymptomatic in children would commonly result in severe post-infection symptoms.” This is supported by a longitudinal cohort study finding that COVID-19 symptoms in children “were not more severe than symptoms from other common respiratory illnesses [during a time when RSV and Influenza were not circulating].”	Kraaijeveld et al.^37^De Hoog et al.^38^
Systematic review of Long-COVID	The often significant rates of persistent physical symptoms in control groups mean that “studies have shown that the pandemic has profoundly impacted society by affecting children’s development through isolation, poverty, food insecurity, loss of parents and caregivers, loss of time in education, and increased stress. COVID-19 pandemic has initiated an explosion of future mental illness . . . The presence of these symptoms in the general population, regardless of COVID-19 status, has been coined long-Pandemic syndrome.”	Lopez-leon et al.^15^
Lancet editorial on Long-COVID	“[The] pandemic is likely to leave long-lasting marks on a generation of children and young people, mainly from indirect effects, including those of school closures, social isolation, and a so-called immunity debt . . . On a population level, the overall impact on children of having had COVID-19 is probably small, and less than the indirect effects of the pandemic.”	Rutter^39^
Adults with Long-COVID		
Systematic review of Long-COVID	“[Long-COVID could be due to] indirect effect on mental health due to post-traumatic stress, social isolation, and economic factors, such as loss of employment.”	Groof et al.^4^
Rapid evidence report on Long-COVID	“Lung opacities, function and exercise capacity are in the normal range.”	Manhas et al.^5^
Systematic review of Long-COVID	“[Long-COVID could be from] pandemic effects on individuals and societies.”	Alkodaymi et al.^6^
Global Burden of Disease collaborators on Long-COVID	“Post-infectious fatigue syndromes have been described for other viruses and bacteria . . . pathology remains largely unknown.”	Global Burden of Disease Long Covid Collaboration^7^
Systematic review of cardiopulmonary exercise testing and Long-COVID	“[Dysfunctional breathing was] unexplained by baseline pulmonary function tests or findings on cross-sectional imaging . . . ventilatory, pulmonary vascular, and cardiac limitations are uncommon, suggesting that direct heart or lung damage (especially given other negative testing results) are not major drivers of exercise limitations.”	Hasiam and Prasad^44^
Narrative review of neuropsychiatric sequelae in Long-COVID	“Pandemic-related stressors have been shown to affect such symptoms as cognition, anxiety, depression, fatigue, and sleep and may play a larger role in generating these symptoms than SARS-CoV-2 infection itself.”	Fontera and Simon^8^
Systematic review of fatigue and cognitive impairment in Long-COVID	“[Long-COVID could be] a form of post-infectious fatigue syndrome, and exhibit phenotypic similarity to ME/CFS, which is often precipitated by an infectious agent . . . may be consequences of chronic stress and/or depression resulting from social and economic challenges of COVID-19, rather than a result of infection.”	Durstenfield et al.^43^
State of the Art review on Long-COVID mechanisms	“[Re: fatigue, a] cross-sectional study found no association between pro-inflammatory markers and long-term fatigue.” “[Re: dyspnea] most individuals who develop long term breathing difficulties post-COVID-19 have no signs of permanent or long lasting lung damage.”	Crook et al.^9^
	“[Re: cognition and mental health] quarantine, isolation, and social distancing also have damaging effect on mental health and cognition . . . pandemic exerted extra unfavorable effect on loneliness, physical activity, and mental health . . . protracted social isolation has resulted in exacerbation of neuropsychiatric and behavioral disturbances, including apathy, anxiety, agitation, boredom, and confusion in dementia patients . . . knowledge of the COVID-19 death toll also has a negative impact on quality of sleep, stress, anxiety, and other negative emotions, and sleep problems have been shown to be associated with COVID-19 related loneliness . . .” Overall, ultimately concluding the cause “remains enigmatic.”	
Systematic review of brain molecular imaging in Long-COVID	“Some authors found extensive areas of limbic and subcortical hypometabolism, whereas others found no metabolic alterations on PET and only minor cognitive impairments (if any) on neuropsychologic assessment.”	Meyer et al.^80^
Narrative review of the neurobiology of Long-COVID	Asserted many “potential underlying mechanisms that could contribute to CNS dysfunction,” relying almost solely on rare small autopsy studies of patients who died of severe acute COVID-19.	Monje and Iwasaki^77^
Narrative review of the neuropsychiatric aspects of Long-COVID	Found “no specific abnormal findings in the blood or cerebrospinal fluid,” that symptoms “are not likely to be caused by persistent infection in the central nervous system,” and that on neuroimaging “most cases do not show any specific visually detectable lesions.”	Kubota et al.^84^
Narrative review of CFS and COVID-19	Found no association between “chronic symptoms, and objective measures of respiratory function, suggesting an alternate mechanism of pathogenesis . . . it will be difficult to separate the impact of pandemic-associated stress from the impact of the infection itself . . .”	Poenaru et al.^149^
Autopsy study with COVID-19	Autopsy studies have found “little evidence of inflammation or direct viral cytopathology outside the respiratory tract [including in the brain].”	Stein et al.^79^
Longitudinal cohort study of COVID-19 sequelae and immunity	An exhaustive search for pathology, including physical exam, neurocognitive testing, biomarkers of organ injuries or autoimmunity, pulmonary function testing, echocardiogram, markers of immune activation, and markers of persistent viral infection found a “lack of objective evidence of tissue damage or organ dysfunction . . . [or] abnormal systemic immune activation or persistent viral infection in participants with [Long-COVID].” Concluded that “the constellation of subjective symptoms in the absence of objective abnormalities on diagnostic evaluation resembles what has been described with other illnesses (CFS/ME, post-infection syndromes, mental health disorders).”	Sneller et al.^83^
Narrative review of Long-COVID^a^	The introduction wrote that “Mechanistic studies are generally at an early stage, and although work that builds on existing research from postviral illnesses such as ME/CFS has advanced some theories, many questions remain and are a priority to address.” The last conclusion sentence wrote that “Diagnostic and treatment options are currently insufficient, and many clinical trials are urgently needed to rigorously test treatments that address hypothesized underlying biological mechanisms, including viral persistence, neuroinflammation, excessive blood clotting and autoimmunity.”	Davis et al.^85^
Systematic review by the Chief Science Advisor of Canada expert advisory panel	The executive summary wrote that “The biological basis for the complex symptoms and conditions seen in PCC remains unknown and represents a major impediment to diagnosis and care of individuals suffering from the condition.” The section on “Underlying Causes” wrote “At present, the underlying causes of PCC remain undefined, and it is unclear why a subset of individuals infected with SARS-CoV-2 develop PCC.” The section on knowledge gaps in causal mechanisms wrote “The mechanism(s) of pathogenesis of PCC and the full disease course from first exposure to clinical phase and recovery is not understood.”	Nemer et al.^86^
Narrative review of Long-COVID	“The pathogenesis of long-COVID is clearly multifactorial and difficult to unravel. It is still not clear what role the persistence of the virus has in different organs, its reactivation, and the long-term immune response.”	Baroni et al.^87^
Systematic review of chest imaging findings in Long-COVID	The abstract concluded that “Although respiratory symptoms belong to the most common symptoms in long COVID patients, this is not necessarily linked to radiologically detectable lung damage.” The discussion also wrote that “A major finding of this systematic review was that although the dominant symptoms in long COVID patients are respiratory, this is not necessarily related to lung imaging abnormalities.”	Bazdar et al.^88^
Systematic review of chest CT findings in Long-COVID	The summary statement wrote that “The prevalence estimates of 1-year chest CT lung abnormalities after COVID-19 are highly heterogeneous among available studies. Although they represent potentially reversible changes, data are insufficient to draw firm conclusions.” The review also discussed that “Given these observations, we believe the alarmism about the risk of pulmonary fibrosis due to COVID-19 pneumonia in post–COVID-19 condition should be dampened.” The conclusion statement was that “Despite changes being mostly represented by non-fibrotic potentially reversible sequelae, the determinants of heterogeneity still remain unknown. This gap represents a substantial limitation that requires caution in data interpretation in the absence of convincing evidence.”	Bocchino et al.^89^
Systematic review of findings in Long-COVID	Found that “Pathology tended to be reported in only a small number of studies, with the exception of lung pathology, which was reported in 26 studies.” The meta-analyses of organ pathology reported that the 95% prediction intervals included 0% for pathology of the pancreas, liver, kidney, or vascular organs, and included ⩽0.5% for pathology of the heart, or neurological organs, all with high heterogeneity (*I*^2^ at least 95%). Lung pathology was reported in 38.9% (3.4, 91.9), with very high heterogeneity (*I*^2^ 99.7%).	Woodrow et al.^71^
Systematic review of sequelae of the COVID-19 pandemic	Found that “Of all organ systems, the respiratory system was most commonly affected with an estimated 55.6% (95% CI 46.8–64.2) of patients experiencing abnormalities in any objective examination results based on 32 studies (6292 participants), including either abnormalities on lung CT (56.9%, 95% CI 46.2–67.3) or abnormal pulmonary function [mostly DLCO] testing (45.6%, 95% CI 36.3–55.0).”	Zeng et al.^90^
Other studies (non-reviews)	Individual studies also had similar statements alluding to the medically unexplained nature of symptoms.	Haddad et al.,^23^ Bull-Otterson et al.,^52^ Al-Aly et al.,^64^ Townsend et al.,^73^ Dennis et al.,^81^ Kremer et al.^82^

CFS, chronic fatigue syndrome; ME, myalgic encephalomyelitis; PET, positron emission tomography.

In response to one of the reviewers, we searched PubMed to prevent missing systematic reviews relevant to structural organ pathology in Long-COVID. First, we searched “Long COVID” OR “Post COVID condition,” AND “systematic review,” from January 1, 2023 to July 14, 2023. This returned 52 citations, and on review of the abstract and full text to detect data on structural organ pathology, 3 were included.^87–89^ Second, we searched “Long COVID” OR “Post COVID condition,” AND “pathology,” AND “systematic review” with the same date restriction. This returned nine citations, and on review of abstract and full text to detect data on structural organ pathology, two more publications were included.^71,90^

a This review has an Almetrics score of 14,526, which puts it “in the 99^th^ percentile (ranked 4th) of the 441,563 tracked articles of a similar age in all journals and the 99^th^ percentile (ranked 1st) of the 38 tracked articles of a similar age in Nature Reviews Microbiology” (see: https://www.nature.com/articles/s41579-022-00846-2/metrics).

### In adults

There are more studies of Long-COVID in adults than in children. Again, a striking feature is that severe study limitations make definitive statements difficult; almost all studies are of moderate or low quality.^40^ Systematic reviews consistently list these common limitations, including attrition bias,^40^ severity of Long-COVID symptoms not defined,^5,6,40,41^ usually no controls or even baseline prevalence of symptoms,^6,8,40–42^ unmeasured confounder bias (e.g., people who had COVID may be vulnerable in ways that explain both why they got COVID and why they went on to have adverse outcomes, with most studies adjusting for few if any covariates),^4,8,40,42–44^ selection bias (e.g., due to many included patients having been hospitalized, online recruitment, non-laboratory-confirmed diagnosis of acute infection, and bias to testing those with access to healthcare and with strong health-seeking behavior),^5,42,43,45,46^ and recall bias due to unblinded self-reporting.^7,41^ Other biases mentioned in individual studies included ascertainment bias (e.g., being more actively monitored after COVID-19),^47,48^ unblinded participants (e.g., who may be more likely to report symptoms after a positive test),^23,49–51^ unblinded investigators (e.g., who may be more likely to document possible post-COVID conditions among cases),^52^ misclassification bias (e.g., often acute COVID-19 was not a laboratory-confirmed SARS-CoV-2 infection, with the denominator not based on seroprevalence),^48,49,52–56^ and possible reverse-causation (i.e., new conditions may be risk factors for more severe acute COVID-19 and may predate the index event).^52^ We should not underestimate the effect of these biases that create a self-fulfilling prophecy (i.e., the more doctors look, the more doctors will find). In addition, the plethora of nonspecific, prevalent, often intermittent/fluctuating/relapsing or new usually self-reported symptoms^9,40,46,57,58^ make it “difficult to operationalize [Long-COVID] in clinical settings”;^59^ in other words, it is

often unclear whether particular symptoms could be attributed to Long-COVID, given the medical complexity and functional limitations of many patients and absence of specific markers for this condition, which could lead to ongoing monitoring, diagnostic testing, and specialist referral . . . [and] other comorbid conditions [and changes in their treatments] with symptoms that could potentially overlap [with Long-COVID] . . . .^59^

For all these reasons, systematic reviews find very high heterogeneity between studies.^4–6,9,40,42,45^

Long-COVID often has a high incidence in studies without control groups. For example, Chen et al. found a global pooled prevalence of 43% (95% confidence interval (CI) 39, 46) among 31 studies (only one study was mentioned as having had a control group in Table 1), higher among hospitalized (54%) than non-hospitalized (34%) cases.^42^ However, this incidence is much lower in studies that had control groups.^23,40,49,56,60,61,62,63^ For example, the Office of National Statistics in the UK found a difference of 1.6% in incidence compared to controls.^49^ A prospective multicenter registry study found persistently poor physical, mental, or social well-being (i.e., moderate to severe impairments across any PROMIS domain) at 3-month follow-up of 39.6% in COVID-19-positive versus 53.5% in COVID-19-negative people, persistently poor mental health (i.e., moderate to severe anxiety or depression) at 3-month follow-up in 21.9% COVID-19-positive versus 27.3% COVID-19-negative people, with more improvement from baseline in the COVID-19-positive participants.^63^ Studies using electronic health records have also found low rates. In the UK, (i) general practice consultation rates for symptoms among previously hospitalized COVID-19 patients compared to controls were <1% higher, except for breathlessness (1.4% higher), and among community COVID-19 cases was <1% increased from baseline, such that “healthcare use was similar to that in the negative control group”^47^ and (ii) compared to controls, according to any of 32 symptoms, Long-COVID was 1.08% higher, according to any of 62 symptoms, it was 1.92% higher, and individual symptoms had very small differences between cases and controls (<0.05% for 49, 0.05–0.10% for 5, 0.1–0.2% for 6, and >0.2% for the two symptoms of pain at 0.71% difference and fatigue at 0.22% difference).^50^ In US healthcare database studies, (i) one new type of clinical sequelae that required medical care was 1.65% higher than in a viral LRTI control group, with non-hospitalized cases having a risk difference well <1%;^48^ (ii) non-hospitalized cases had small absolute risk differences compared to controls of <2% for all symptoms except respiratory signs/symptoms at 2.85%, and hospitalized cases usually at <2% for all symptoms except for malaise/fatigue at 3.65% compared to influenza controls;^64^ and (iii) non-hospitalized cases had shortness of breath incidence 0.4% higher than controls, with other symptoms higher in test-negative patients, and hospitalized cases had differences from controls ⩽1% for symptoms except for shortness of breath at 5% and fatigue at 2.1%.^30^ A UK healthcare worker follow-up study at three hospitals found no difference in cardiovascular findings (cardiac structure, function, magnetic resonance imaging (MRI) tissue characterization, and biomarkers) between COVID-19 cases and uninfected controls, writing that this “provides societal reassurance for the cardiovascular health of working-age individuals with convalescence from mild SARS-CoV-2.”^65^

Systematic reviews have also suggested a high incidence in studies without controls,^4–6,8,40,42,45^ but small differences when compared to control groups.^6,7^ Alkodaymi et al. found “only 6 studies with comparator group of COVID-19 negative patients . . . only two were rigorously designed,”^42^ and these studies found a higher risk of mood disorder and anxiety,^66,67^ but a lower incidence of most symptoms (sore throat, cough, body ache/muscular pain, nasal symptoms, headache, abdominal pain, or nausea/vomiting) in the case group,^60^ no difference in neurological or cognitive deficits among healthcare worker cases and controls,^67^ and no difference in health-related quality of life domains among hospitalized cases and controls.^68^ The Global Burden of Diseases Collaborators found at least one cluster of Long-COVID in 6.2% of cases (fatigue cluster 3.2%, cognitive cluster 2.2%, and respiratory cluster 3.7%) compared to controls or to status prior to COVID-19 infection.^7^ In a review of UK studies, Thompson et al. found long-COVID among 7.8–17% of cases (without a control group), but when limited to people with symptoms that “limited day-to-day function,” the rate was lower at 1.2–4.8%.^41^ Also of note, studies suggest much lower odds of Long-COVID during the Omicron variant wave.^51,69,70^ Another systematic review found the risk difference (95% prediction intervals) for Long-COVID compared to controls was 13.9% (−16.2, 43.9), compared to controls in community-based samples 10.1% (−12.7, 32.8), and compared to controls in community-based samples assessed as at low risk of bias 4.8% (−13.2, 22.7).^71^

Among COVID-19 cases, risk factors for Long-COVID in systematic reviews included female gender,^5,7–9,41, 42,45,72^ dyspnea in the acute phase or asthma/chronic-lung-disease/chronic-dyspnea,^5,41,42,72^ previous psychiatric diagnosis (including prepandemic psychological distress),^5,8,41,72^ severity of acute COVID-19 (including number of acute symptoms^42^ and hospitalization,^7,42,43,72^ especially in intensive care),^5,7–9,42,43,72^ and underlying comorbidity.^8,42,45,72^ Individual studies have also emphasized psychiatric diagnoses as risk factors, for example, preexisting fibromyalgia, anxiety, depression, migraine, irritable bowel syndrome (IBS), eating disorder, or back pain;^50^ and preexisting diagnosis of depression or anxiety.^73,74^ Although intensive care admission has been identified as a risk factor, there appeared to be no difference from other intensive care controls.^75^ Pre-infection poor sleep health was also an important risk factor.^76^ An interesting finding was that in a US healthcare plan study, cases also had an increased risk of new clinical sequelae that required medical care 30 days *before* the index COVID-19 diagnosis date.^48^

As mentioned above, many reviews hypothesized tissue damage as the cause of Long-COVID.^4–9,77^ A recent review in Scientific American went so far as to claim that Long-COVID is a neurological disease due to some combination of thrombotic events, central nervous system inflammation and autoimmunity, and persistent viral infection/proteins in the brain and that treatments may include IVIG, rituximab, corticosteroids, beta-blockers, and amphetamine/dextroamphetamine.^78^ However, many reviews also reported that symptoms largely remain unexplained,^4–9,23,43,45,53,64,71,73,77,79–90^ as summarized in Table 2.

The most frequent symptoms of Long-COVID have included shortness of breath/dyspnea, fatigue/exhaustion, sleep disorders/insomnia, cough, cognitive problems (with concentration and memory), and psychiatric problems (anxiety and depression).^4–6,40,42^ These symptoms have been suggested to occur in overlapping clusters of persistent fatigue with bodily pain or mood swings, cognitive problems commonly referred to as “brain fog,” and ongoing respiratory problems.^7,50^ Despite studies not determining the severity of these symptoms, some have made alarming claims about them. For example, “[fatigue is] unrelenting exhaustion and a constant state of weariness that reduces a person’s energy, motivation, and concentration,”^9^ and “millions of economically active people may be disabled by Long-Covid” stating that fatigue was “a feeling of utter exhaustion, energy drain, or bodily dysfunction that is not necessarily triggered by exertion and is not always relieved by rest.”^57^ Another review warned of “an alarming picture of an emerging neurological health crisis.”^77^ A recent narrative review of Long-COVID claimed that COVID-19 “can severely damage multiple organs, including the nervous system” and that “COVID-19 has significant long-term effects on the nervous system.”^84^ A Scientific American paper asserted that patients have “extreme fatigue,” “add up to millions more people affected—and potentially disabled,” and “[Long-COVID] could last many years.”^78^ We believe this exaggeration is counter-productive, as will be discussed later.

## Long-pandemic effects

Given that a major risk factor for Long-COVID included worse pre-COVID mental health, it is important to emphasize the adverse effect that the pandemic response is having on the population.

In children, the American Academy of Pediatrics called a national emergency in children’s mental health, with an “escalating mental health crisis due to physical isolation, ongoing uncertainty, fear, and grief,” including increased mental health emergencies, suicide attempts, depression, anxiety, trauma, loneliness, and suicidality.^91^ In Canada, The Hospital for Sick Children similarly reported higher depression and anxiety in children associated with time spent online learning, less participation in sports and extracurriculars, and increased screen time.^92^ The report noted “significant and sustained mental health effects that the public health mitigation strategies and school closures have had on children . . . Kids need school, they need their friends, and they need to have fun.”^92^ Systematic reviews have documented high rates of anxiety and depression symptoms,^93–97^ noting that “school closures and social lockdown . . . were associated with adverse mental health symptoms (such as distress and anxiety) and health behaviors (such as higher screen time and lower physical activity),”^93^ and that these rates are double baseline rates of anxiety and depression.^94^ These mental health effects were predicted in a prepandemic systematic review in children that found “social isolation and loneliness increased the risk of depression, and possibly anxiety at the time at which loneliness was measured and between 0.25 and 9 years later.”^98^ A study confirming these more severe internalizing mental health problems in youth also found that, compared to carefully matched peers before the pandemic, after pandemic shutdowns, there was maladaptive neurodevelopment on MRI with reduced cortical thickness, larger hippocampal and amygdala volumes, and more advanced brain age (findings typical of previous cohorts that had experienced significant adversity during childhood).^99^ A systematic review also found that “considerable disruption in the lives and routines of children, adolescents, and families” have led to an approximately 52% increase in screen time.^100^ Studies in the United States have found increased rates of mental health conditions including eating disorders, depression, anxiety, and sadness.^101–114^ Studies from around the world have found markedly increased rates of eating disorders,^105–108^ likely due to increased anxiety and “a combination of social isolation and school closures [that] has disconnected patients from protective factors . . . reduction of extracurricular activities, school routine and peer relationships have created room for eating disorder cognitions to intensify.”^105^ Several studies have also found reduced physical activity in children during the pandemic,^92,93,103,109–111^ which is a strong risk factor for depression.^112^ Not surprisingly, the pandemic has been associated with increasing body mass index,^113,114^ and reduced cardiorespiratory fitness in children.^115^ As editorialists commented, “when we close schools, we close their lives.”^116^

In adults, highly prevalent adverse mental health, including stress, anxiety, depression, substance use, suicidal ideation, and loneliness, have been documented in systematic reviews^117,118^ and individual studies in the United States^119^ and the UK.^120^ The COVID-19 mental disorders collaborators identified being female as a risk factor, and commented that this may be due to women being more likely to be affected by the social and economic consequences of the pandemic response (e.g., having additional carer and household responsibilities due to school closures or unwell family; more likely to be financially disadvantaged due to lower salaries, less savings, and less secure employment; more likely to be victims of the increased domestic violence).^118^ In addition, government covert psychological strategies used to induce fear in the population likely contributed, with people being “bombarded with fear-inducing information,”^121^ aiming to increase “the perceived level of personal threat” which “ultimately it [sic] backfired because people became too scared.”^122^ Taylor has coined the term “COVID Stress Syndrome” that describes this fear, including fear of becoming infected, of coming into contact with fomites or foreigners, and of socioeconomic consequences, often with compulsive checking and reassurance seeking, and traumatic stress symptoms.^123^ Of note, an unhealthy lifestyle had a dose-dependent association with Long-COVID, and this included physical activity, diet quality, and adequate sleep, factors that were adversely affected by the pandemic response.^124^

It is worth mentioning that, even prepandemic, stress and adverse mental health were associated with inflammation and neuroinflammation, making the direction of causality difficult to determine.^8,125–129^ Brusaferri et al. found in pandemic patients without having had COVID-19, “novel evidence of elevated neuro-inflammatory markers in cortical and subcortical regions . . . implicates neuroimmune activation as a possible mechanism underlying the non-virally-mediated symptoms experienced by many during the COVID-19 pandemic.”^128^ Reviews have written that if inflammation is found, this is “consistent with decades of psychoneuroimmunology research in patients with anxiety disorders, depression, and traumatic stress-related disorders,”^8^ “inflammation and immune dysregulation may link psychological distress with post-COVID-19 conditions [e.g., distress is associated with chronic systemic inflammation, and “mental health disorders are associated with chronic low-grade inflammation and microglia activation” in the central nervous system],”^125^ “psychosocial factors are also very important in regulating our immune system [e.g., immune abnormalities are found in chronic fatigue syndrome (CFS), fibromyalgia, chronic pain, depression and other mental health disorders, with increased peripheral inflammation and activation of glial cells with neuroinflammation],”^126^ and suggesting that maladaptive behavioral responses are actually causing the abnormal immune findings.^127^ Poor sleep health, which increased during the pandemic, is associated with chronic low-grade inflammation and immune abnormalities, COVID-19 risk and severity, and Long-COVID.^76^ Anxiety and depression have been found to be associated with more severe acute respiratory infections, including COVID-19 (i.e., hospitalization, intensive care, and mortality), and it is possible this is mediated by the associated immune abnormalities.^130^ Psychosocial stress is a strong modifiable risk factor for stroke (with higher locus of control at home or at work being an effect modifier lowering this risk), also possibly mediated by neuroinflammation.^131^ Compatible with this is that social disconnection (e.g., social isolation and loneliness) is “a key determinant of health” with an effect on all-cause mortality “comparable in magnitude with that of smoking (15 cigarettes/day) and high levels of alcohol consumption (6 drinks/day).”^132^ Another systematic review that found social isolation and/or loneliness associated with increased all-cause mortality suggested a mechanism may be that they “lead to activation of the hypothalamic-pituitary-adrenocortical axis . . . [which] affects a wide range of physiological functions, including . . . metabolism and inflammatory control [with pro-inflammatory immune response] . . . .”^133^

## Functional somatic disorders (Diagnostic and statistical m﻿anual of m﻿ental disorders (DSM-V) somatic symptoms and related disorders)

We use the term “functional” to describe these disorders, and the term “medically unexplained” to describe the associated symptoms. These terms are not satisfactory and can be misleading and used pejoratively; however, we use them as commonly used in the medical literature.^134^ The term “functional” is misleading because it can imply that all is “functioning normally” in the body so that symptoms are “all in the head,” which mistakenly suggests that other so-called structurally caused symptoms are not also *experienced* “all in the head” and that not all symptoms are perceived through the same common pathways (“reflecting the [complex interactions between, and] integration of bodily and brain functions and dysfunctions”), as explained later.^134^ The term “medically unexplained” is misleading because it “implies that explanations that involve psychosocial or cultural factors are not part of medicine,” and as explained later, these in fact do have an organic brain function explanation.^135^

### Somatic symptom disorder

SSD describes a patient with persistent physical symptoms (that may include bodily complaints of pain, fatigue, and perceived disturbances of organ functions) that are distressing and/or result in significant disruption in daily life.^136–139^ These symptoms are accompanied by disproportionate and persistent thoughts (about the seriousness), feelings (high health anxiety), and/or behaviors (excessive energy or time devoted to health concerns) related to them.^136–138^ These are real experienced symptoms that often cause significant disability.^136–138^ These are also medically unexplained symptoms (MUS), in that there is no explanatory structural or other pathology.^140,141^ Both children and adults with somatic symptoms and related disorders have frequent healthcare consumption (sometimes related to under-recognition) and disability.^142,143^

An SSD is surprisingly common,^137–139^ with a systematic review finding a general population (using self-report) mean frequency of 12.9% (95% CI 12.5, 13.3), and a non-specialized general medicine setting mean frequency of 35% (95% CI 33.8, 36.2).^137^ Reviews have identified predisposing, precipitating, and perpetuating factors for SSD. Predisposing factors include childhood adversity (including parental ill health), stress, female gender, depression, and anxiety (including illness worries, catastrophizing, and fear); of note, these are not required to make the diagnosis.^135–141^ Precipitating factors (triggers) include acute illness (viral or other infection, physical trauma, or illness) and psychosocial trauma (negative life events, environmental events such as terrorist attacks).^135–141,144^ Many diverse infections have been associated with so-called unexplained post-acute syndromes with “remarkably consistent” “core symptoms centering on exertion intolerance, disproportionate levels of fatigue, neurocognitive and sensory impairment, flu-like symptoms, unrefreshing sleep, myalgia/arthralgia, and a plethora of nonspecific symptoms.”^144^ Perpetuating factors (context amplifiers) include poor physical and social activity, poor sleep hygiene, missed/late diagnosis (often with medical uncertainty, inappropriate treatments, and frustrations with care), unhelpful cognitions (e.g., fear that exercise is damaging, that there is a missed physical cause for symptoms, or symptom focusing), and social reinforcement (e.g., by the wider social circle and media).^135–141^ Suggested treatment is multidisciplinary and includes explaining the diagnosis of a somatic symptoms disorder through education of the patient and caregivers (including the disorder of brain function explanation to be discussed later), graded physical therapy/rehabilitation, and cognitive behavioral therapy, with an emphasis changed from cure to care and coping, focusing on perpetuating factors that affect overall functioning.^136,138^ Prevention and treatment would also include managing depression and anxiety (including reducing illness worries and fear).

### Related disorders (syndromes)

These are SSD that consist of certain medically unexplained persistent physical symptoms that have been named. Fatigue and pain cluster in syndromes of IBS, CFS, fibromyalgia, and some chronic pain syndromes, and cardiorespiratory symptoms cluster in non-cardiac chest pain or hyperventilation.^140^ CFS is associated with debilitating fatigue, malaise, headaches, muscle/joint pain, nausea, disrupted sleep, dizziness, and cognitive difficulties.^145^ The same predisposing, precipitating, and perpetuating factors as in SSD have been identified.^139–141,145,146^ The trigger has often been a recent viral (or other) infection.^144,147,148^ There are no specific diagnostic physical signs or biomarkers, and no consistent cytokine, cellular, auto-antibody, mitochondrial, or neuroimaging findings.^149^ A systematic review found that “the proportion of patients with one unexplained clinical condition meeting criteria for a second unexplained condition was striking” with about 30–80% overlap among CFS, fibromyalgia, IBS, multiple chemical sensitivity, temporomandibular disorder, tension headache, interstitial cystitis, and post-concussion syndrome.^150^ Another systematic review found CFS and fibromyalgia coexist in 47.3% of cases.^151^ These functional syndromes shared commonalities in symptoms (e.g., fatigue, pain, abdominal distension or bloating or pain, headache), disability out of proportion to physical examination findings, inconsistent demonstration of laboratory abnormalities, and an association with “stress” and psychosocial factors.^150,152^ Treatment suggestions are similar to SSD in general, focusing on explanation and education, physical therapy/rehabilitation, and cognitive behavioral therapy (aiming to change maladaptive thoughts and behaviors).^127,145^

An older study is particularly interesting.^153^ Of those suffering from environmental hypersensitivity disorder, 90% reported suffering from at least one other so-called “fashionable” (i.e., media popularized) condition, including food allergy, candidiasis hypersensitivity, post-infectious neuro-myasthenia, fibrositis, and temporomandibular joint syndrome.^153^ Patients had multiple, vague, nonspecific, ill-defined, and generally common symptoms (e.g., fatigue, nausea, dizziness, respiratory symptoms, poor concentration and memory, headaches).^153^ The author suggested that sociocultural context, including “intense media interest and hyperbole” and “illness-behavior role models” influenced the attribution of cause and exaggerated worry over health.^153^ In addition, perpetuating factors were suggested to include becoming active advocates of the disease, propensity for self-diagnosis, and achieving a “scientific aura” provided by media, support groups, and healthcare providers, such that the “disorder becomes their identity.”^153^ Of note, some have considered chronic Lyme disease a functional syndrome, with risk factors identified to include depression, negative affect, catastrophizing pain, anxiety, and past traumatic psychological events.^154–157^

### Functional neurological disorders

FNDs are common and involve significant, genuinely experienced neurological symptoms without structural pathology that cause considerable distress and disability.^158–165^ Disorders are commonly motor (including tremor, dystonia, tics, paralysis, abnormal gait, and/or paroxysmal non-epileptic seizures) and can be sensory.^159–164^ Cardinal features include that symptoms fluctuate in severity, waxing and waning over time, decrease with distraction and increase with body-focused attention, and movements are made with excessive effort and fatigue, with positive neurological signs that demonstrate internal inconsistencies (i.e., FND is not a diagnosis of exclusion).^162–164,166^ FND symptoms often coexist with other persistent medically unexplained physical symptoms including dizziness, chronic pain, fatigue, sleep disturbance, memory symptoms, and dissociative symptoms.^160–162,164^ In adults, FNDs also often coexist with other related disorders (syndromes) including IBS, fibromyalgia, chronic pain, and cardiorespiratory symptoms.^160,161^ Associated anxiety and depression are common.^160–162,164,165^ Similar (to SSD) predisposing, precipitating, and perpetuating factors have been identified.^159,160,162–167^ Panic attacks and autonomic hyper-arousal are included as predisposing and precipitating factors for FNDs, and are likely similar to fear and anxiety being included for SSD.^160,162^ Treatment is recommended to be multidisciplinary and to focus on giving and explaining the functional diagnosis, emphasizing the condition is potentially reversible, providing physical therapy/rehabilitation, cognitive behavioral therapy, and follow-up.^158,160,162^ In explaining the diagnosis to patients, it is suggested not to simply emphasize normal test results, say “there is no neurological disease,” or focus prematurely on psychiatric comorbidity (i.e., don’t turn a risk factor into the “cause,” and don’t suggest a mind–body dualism).^158,160,163^ If not explained properly, often the patient hears “this is all in your head,” disrupting patient–clinician trust and further complicating recovery.

The FND variant functional cognitive disorder (FCD) is particularly interesting because patients often describe “cognitive fog” or “brain fog” (terms used well before the COVID-19 pandemic).^144,168–170^ This common disorder, estimated to comprise at least one-quarter of memory and cognition clinic referrals, is characterized by subjective memory impairment, attention and concentration difficulties, and common co-occurrence of multiple functional symptoms, in the absence of underlying brain structural pathology.^168,170,171^ The positive diagnostic feature is internal inconsistency, where “functions that remain easy and automatic become difficult when attention is focused towards them.”^170^ The cognitive symptoms may be due to a lack of attentional reserve, as evidenced by being susceptible to distraction, with slow information processing during periods of excessive attention toward the body.^169^ “Similar cognitive symptoms [forgetfulness, distractibility, word-finding difficulties] . . . [are] correlated with pain in Fibromyalgia and with mental or physical exertion and fatigue in CFS . . . Evidence does not support the existence of separate cognitive disorders in CFS, Fibromyalgia, and FND.”^169^ So-called brain fog has been described in association with a wide range of illnesses, drugs, and behaviors, including Long-COVID.^172^

### Long-COVID as a form of functional disorder

Some studies report findings that suggest Long-COVID may be a functional syndrome. Wang et al. found that preexisting depression, anxiety, worry about COVID-19, perceived stress, and loneliness, in a dose-dependent manner, were stronger risk factors for Long-COVID than other established risk factors.^125^ A Norwegian study found that, in non-hospitalized 12- to 25-year-olds, SARS-CoV-2 positivity was not associated with the development of Long-COVID or post-infective fatigue syndrome at 6 months, while baseline symptoms severity, low physical activity, loneliness, and prior negative life events were.^173^ Another study found that, among 790 COVID-19 patients who survived hospitalization, life stressors were the strongest independent predictors of prolonged symptoms.^174^ Somatic symptoms among Chinese students have correlated with concern regarding the threat to life and health from COVID-19.^175^ A single-center study of Long-COVID patients referred to a neurology clinic found that 0/49 (0%) had specific brain MRI findings, 32/50 (64%) met DSM-5 criteria for SSD, and in the remaining 18/50 (36%) patients “SSD was considered possible given the high score on diagnostic scales.”^176^ In this study, patients described feeling significant anxiety, social isolation, fear of infecting relatives, and fear of dying during the acute infection, and 38% had a premorbid functional disorder preceding the infection.^176^

Willis and Chalder explicitly suggested that Long-COVID may be an SSD.^177^ They suggested that pandemic effects “create a ‘perfect storm’ for the development of persistent physical symptoms,” contributing to predisposing (e.g., psychological distress, stress, anxiety, depression, inactivity, social isolation, adverse media exposure), precipitating (e.g., acute COVID-19 symptoms), and perpetuating (e.g., beliefs of a serious prolonged illness conveyed by the term “long-hauler” and medical and media portrayal of serious consequences and prolonged recovery) factors.^177^ Another group suggested that “a new paradigm is needed to explain long COVID” and “it is time to break taboos based on a dualistic understanding of physical versus mental illness and bring in existing knowledge about functional somatic symptoms to provide improved explanations and treatments.”^178^

Several authors have reported that many Long-COVID patients (between 27% and 45%) meet the criteria for CFS.^179–182^ A systematic review found Long-COVID descriptions to have similar neurological symptoms to CFS, including problems thinking, remembering, or concentrating, dizziness, fatigue, headaches, muscle or joint pain, and sleep problems.^181^ A systematic review of 52 studies found the incidence of CFS in Long-COVID to be 45.2%.^183^ In Long-COVID, subjective cognitive complaints were common without abnormal neuropsychological battery scores, and altered dyspnea perception occurred without abnormal lung function.^179^ In a single-center study of Long-COVID patients referred to a neurology clinic, 45/50 (90%) met the criteria for CFS, 48/50 (96%) had symptoms compatible with FCD, all had an absence of specific MRI changes, and in the 15 that had a neuropsychological assessment, only mild impairment of attention was found.^176^ Another study found that 6/15 (40%) Long-COVID patients met screening criteria for fibromyalgia.^184^

Many studies have reported FND during the pandemic. A systematic review found that Long-COVID patients can have similar neurological symptoms to those found in FND, including dizziness, dysphagia, facial pain and spasms, fatigue, headaches or migraines, olfactory symptoms, depression, movement disorders pain, and sleep problems.^181^ Increased presentations (up to threefold) of functional motor disorders and other FND have been reported in children and adults, attributed by study authors to pandemic psychological stressors related to social isolation, financial strain, loneliness, anxiety, depression, and mobility restriction.^185–187^ Several studies have documented a marked increase during the pandemic in functional tick-like behaviors (a form of FND) in children and adolescents, especially females, often associated with other somatic symptoms.^188–190^ This functional disorder is believed to have occurred with the pandemic-associated surge in social media and digital technology use (i.e., viewing of social media content involving tic-like attacks) combined with increased stress and isolation associated with imposed pandemic restrictions (i.e., lockdowns and mental health deterioration).^188,189,191^ Those with functional seizures (paroxysmal nonepileptic seizures) reported increased frequency during the pandemic.^192^

Some side effects of vaccines for COVID-19 have been recognized as precipitating functional disorders not pathologically due to the vaccine.^193–195^ The WHO has coined the term “neurological immunization stress-related responses” to describe this phenomenon due to “pandemic stress, feelings of uncertainty about COVID vaccination, normal transient physical symptoms, and discomfort after vaccination.”^193^ Acknowledged risks included predisposing (e.g., increased somatic attention from checking for signs of COVID and threat-related hypervigilance, abnormal expectations/beliefs, fear and distrust, widespread information in the media, stress, anxiety), precipitating (e.g., pain/myalgia from vaccination), and perpetuating (e.g., diagnostic delay and incorrect diagnosis and treatments) factors.^193,194^ Surely similar points, by analogy, can be made about Long-COVID.

## Mass sociogenic illness

We will include the unfortunate term “mass hysteria” when directly quoting from some sources. The term is unfortunate as it can be interpreted pejoratively, and suggests mental illness. As will become clear below, we intend neither.

### Past exposures

Mass functional illness has been described for centuries across varied cultures.^189,196–200^ Symptoms have included breathlessness (e.g., hyperventilation, shortness of breath, tight chest, cough), nausea and vomiting, headache, dizziness and light-headedness, weakness, fatigue, diffuse musculoskeletal pain, sleep disturbance, and neurological symptoms (e.g., concentration and memory complaints).^196–198,200^ The precipitating event is belief in an environmental cause, with a dramatic emergency response, creating extreme stress and fear of a perceived often unpredictable (or inescapable) threat; this threat often reflects the dominant sociocultural concerns of the time, such as environmental toxicity or infectious diseases.^189,196–200^ Predisposing factors have been identified to include female gender, anxiety (e.g., checking behavior, engaging in preventive and avoidant behaviors), depression, psychological distress (e.g., fear induced by repeated extensive negative media coverage), and unhealthy behaviors (e.g., unhealthy eating, lack of exercise, disordered sleep, lack of socialization).^196–198^ Perpetuating factors involve social contagion, including physical or visual proximity to others who are ill (especially involving reuniting with the group affected), the excitement induced by the emergency and media response (e.g., collective anxiety, stress, and fear), and clinically labeling the illness and providing preferential medical care for it.^196–198,200^ The perceived threat of terrorism or exposure to a poison/toxin or infection has been a common precipitant in modern times.^189,196–201^ Many outbreaks of mass sociogenic illness in the past have been confined to a small group of people, but social (and other global) media “breaks the geographical barriers that typically confine such symptoms.”^189^ Mass sociogenic illness is “exacerbated and self-reinforcing when the negative information comes from an authoritative source, when the media are politicized, and social networks make the negative information omnipresent.”^200^ It has been suggested that the contribution of digital media and the internet to anxiety and emotional contagion may contribute to a global form of mass sociogenic illness as, “what are temporarily, locally limited, isolated outbreaks of mass hysteria, the state may convert into a global mass hysteria for an extended period of time.”^200^

Some authors we believe gave prescient advice about these syndromes (see Table 3), and have suggested that de-escalating fear with clear information is paramount for treatment.^197,198,202^

**Table 3.** Some quotations that merit emphasis, from papers describing mass sociogenic illness prior to the COVID-19 pandemic.

Source	Quotation
Boss et al.^197^ Regarding mass sociogenic illness (at that time, unfortunately, labeled “epidemic hysteria”).	“[D]ata are usually collected in an emotionally charged environment from persons who may have biased perceptions about the nature of the outbreak . . . biases against the acceptability of psychogenic illness among health professionals as well as the public frequently leave us unwilling to even consider the possibility of epidemic hysteria . . . Epidemiologists may not be aware that an epidemic form of hysteria exists, that it might be the sole cause of the illness under investigation, or that it might be operating in conjunction with other diseases . . . .”
Balaratnasingam et al.^198^ Regarding mass sociogenic illness (at that time, unfortunately, labeled “mass psychogenic illness” or “mass hysteria”).	“[D]uring times of threat, the anxious public needs to feel reassured and protected, and people look to authority figures to take control and provide that reassurance. Undoubtedly, the key responsibility of public health agencies during an epidemic of mass psychogenic illnesses is to deal with the fear and anxiety caused by the threat. The circulation of realistic and practical information by the government and media . . . A planned, well-coordinated, strategic approach will help reduce societal vulnerability to mass hysteria and limit the ‘contagiousness’ of such an event.” “[H]ow governments, medical communities, and the media aid society in responding to this fear may have significant impact on the degree to which future presentations of mass hysteria occur and whether or not these are managed successfully.”
Raker et al.^202^ Regarding physical symptoms after Hurricane Katrina	“[U]nlike other disasters, the pandemic is not geographically bounded . . . Officials need to prioritize minimizing lapses in medical care and medication access . . . Public health messaging should attempt to assuage anxiety and provide tips for overcoming fear . . . provide supplemental health services to those who are bereaved by or experiencing major fear and anxiety due to the pandemic.”
Jones et al.^208^ Regarding war syndromes	“[I]t appears therefore culture may play less of a part in determining symptom patterns than has been suggested. Its main impact may relate to the ways that physicians categorize and interpret functional somatic presentations, and the ways that patients act on and explain their symptoms. Thus, culture can often condition a novel medical explanation that satisfies most of society at a particular time . . . .”
Ismail et al.^29^ Regarding gulf war syndrome	“[Gulf War Syndrome] sufferers with symptomatic distress, in the face of no convincing medical explanation, tend to conduct their own ‘search for meaning’ and can attribute their illness to a variety of possible and plausible causes including viruses, immune system dysfunction, diet, chemicals, or even buildings.”

### Past pandemics

The “Russian Influenza” pandemic of 1889–1892 was associated with widespread dread (especially of feared respiratory complications) and media focus, such that the “dread could itself become a ‘nervous’ symptom of the disease.”^203^ “Post-influenza varied from lethargy to lassitude, to more serious conditions such as depression and neurasthenia [notoriously vague and amorphous],” such that “somatopsychic aspects of the disease tended to blur the lines between organic physiological processes and psychogenic categories.”^203^ Symptoms were diverse and unpredictable, and convalescents were “plagued with mysterious and erratic symptoms and chronic illnesses” that were given many names including neurasthenia, nerve exhaustion, and prostration.^204^

There is evidence that other influenza pandemics had similar post-infectious outcomes. The 1918–1919 pandemic was followed by symptoms including “loss of muscular energy” and debilitating lethargy, “nervous complications” and “apathy and depression,” and restlessness or sleeplessness.^205^ Spanish flu survivors “reported sleep disturbances, depression, mental distraction, dizziness, and difficulties coping at work.”^206^ A study from Norway during the 2009 influenza H1N1 pandemic found being diagnosed with influenza infection to have an adjusted HR 2.04 (1.78, 2.33) for CFS.^148^ There are 12 reports of outbreaks of CFS since 1934, with a systemic syndrome of excessive fatigue, myalgias, headaches, low-grade fever, other constitutional symptoms, and neuropsychological changes (including forgetfulness, difficulty thinking, inability to concentrate).^207^

### War syndromes

Post-combat disorders (apart from Post-Traumatic Stress Disorder) are common and have been labeled variously as Soldier’s Heart, Irritable Heart, Disordered Action of the Heart, Rheumatism, Shell Shock, Effort Syndrome, Non-Ulcer Dyspepsia, Toxic Neurasthenia, and Gulf War Syndrome.^208^ These “war syndromes” have all included overlapping clusters of common nonspecific multisystem MUSs of fatigue, weakness, sleep difficulties, headache, muscle ache, joint pain, problems with memory, attention and concentration, nausea and other gastrointestinal symptoms, anxiety, depression, irritability, palpitations, shortness of breath, dizziness, sore throat, and dry mouth,^208,209^ and often overlapped with other named functional syndromes including CFS, fibromyalgia, multiple chemical sensitivity, and IBS.^209^ Precipitating factors included the traumatic experience of war, described as “man’s reaction to adversity.”^201^ Predisposing factors identified have included anxiety, depression, and “popular health fears and limitations of medical science [thus conveying “a sense of seriousness”].”^208,209^ Perpetuating factors have included having many investigations, media stories (including the internet), and the secrecy associated with the military (likely inducing more fear).^208,209^

We give what we consider some prescient descriptions in Table 3.^208,209^

### The COVID-19 pandemic

The increase in FND during this pandemic has been suggested to reflect mass sociogenic illness by several authors.^148,185,189,190^ The reach of these disorders has been exacerbated by social media (e.g., so-called TikTok Tics).^185,190,210^ The pandemic response affected the mental health of many people creating widespread anxiety, fear (with anticipated negative impacts), depression, helplessness, adverse experiences, and social isolation without access to supports, again suggested to exacerbate the global reach of these disorders.^189,190,210^

Bagus et al. alluded to Long-COVID as a mass sociogenic illness, and suggested that with “the digital age of global mass and social media, the possibility of global mass hysteria exists . . . [and] can cause real symptoms in a self-fulfilling prophecy . . . anxiety and fear contribute to this process . . . both media and the state may actively contribute to the contagion of fear [e.g., stressing breathing problems, the possibility of unknown long-term irreversible health damage] . . . .”^200^ This suggests that treatment would include reducing stress and fear, and encouraging exercise, socializing, and distractions.^200^ Importantly, they suggested that “negative information which is spread through mass media repetitively can affect public health negatively in the form of nocebo effects and mass hysteria.”^200^

### Nocebo effects

Nocebo effects occur when cognitive expectancy of an anticipated negative future outcome causes that very physiologic negative outcome to occur. Nocebo effects are “powerful, pervasive, and common in clinical practice” and include phenomena such as side effects associated with placebo treatment (known to occur in around one-quarter of people).^211^ Effects include any MUSs such as pain, dyspnea, and even measures of inflammation.^211^ Some symptoms turn out to be preexisting symptoms that were previously ignored or dismissed.^211^ Nocebo effects are exacerbated by anxiety, psychological distress, verbal suggestion (e.g., how a medication is framed), learning (e.g., anticipation based on the experience of others, modeling, reports in mass media and lay press, and social observation), and relationship with the clinician (e.g., worrisome information, pessimistic expectations, social messaging, and therapeutic milieu).^211^ A randomized study found that patients with MUSs in the positive frame (i.e., those given a firm diagnosis and told confidently they would be better in a few days) had much better outcomes on follow-up than the negative frame (i.e., those told the doctor cannot be certain what is the matter with them) group, suggesting that “[the doctor] is the placebo [or nocebo] and his/her influence is felt to a greater or lesser extent at every consultation.”^212^

FND has been suggested to share mechanisms with the nocebo effect, including a functional mechanism (i.e., no organ pathology, with inconsistent waxing and waning of symptoms), “maladaptive prior expectations that are reinforced via attention, stress and anxiety,” and “prior beliefs, negative expectations, heightened attentional focus” sometimes exacerbated by “negative interactions with their doctors, perceived poor treatment, and sensations of feeling abandoned.”^213^ Such is the power of negative suggestion, with social influences and learning, and dissemination of negative information creating self-fulfilling mechanisms for symptoms.^213^

Several studies of Long-COVID suggest that nocebo effects are occurring.^23,24,214,215^ Matta et al. found that belief in having been infected (i.e., self-reported infection) had odds ratio (OR) ranging from 1.39 to 16.37 for persistent symptoms—that is, belief was associated with persistent symptoms to a similar extent among participants with negative and positive serology results.^214^ Having had confirmed infection by laboratory testing was associated only with persistent anosmia, suggesting “symptoms may not emanate from SARS-CoV-2 infection per se.”^214^ Some self-identified Long-COVID support group surveys have also found that symptoms are similar in those having had confirmed and unconfirmed infections, except for loss of smell and taste.^54,55^ Similarly, Rouquette et al. found that COVID-19-like symptoms were associated with long-term depression and anxiety after illness, while seropositivity for SARS-CoV-2 was not.^216^ Liu et al. found that perception of (subjective) cognitive deficit during acute COVID-19 was associated with later Long-COVID, suggesting “an affective component to Post-COVID-19-condition in some patients.”^217^ Haddad et al. found that “the number of moderate or severe persistent symptoms reported by individuals (in both an exposed uninfected group and an infected group) was associated with the number of moderate or severe persistent symptoms reported by their household members [i.e., prolonged symptoms tended to cluster within families],” and that “parents who reported their own health status at T1 was worse or much worse than before the pandemic were around 3-times more likely to report that their child had symptoms that persisted until T2.”^23^ The authors noted that in several other conditions, including chronic pain, CFS, and fatigue, “symptom measures in children are associated with parents’ symptoms, stress, and/or parenting behavior.”^23^ Bertran et al. found that in both SARS-CoV-2 PCR-positive and PCR-negative adolescents, parents having Long-COVID increased the risk of the adolescent having Long-COVID by an adjusted OR 1.74 (95% CI 1.54, 2.01; absolute risk 10.7% higher) and an adjusted OR 1.92 (95% CI 1.50, 2.46; absolute risk 10.7% higher), respectively.^215^ The authors hypothesized that parental Long-COVID “increases the focus of attention on symptoms and results in increased frequency of reporting by children of parents with ongoing COVID-19 problems.”^215^ Sorg et al. found that clustered CFS symptoms or substantial fatigue among children and adolescents unaware of their previous seropositive infection status were no different from seronegative controls, although this was not the case when including those who were aware of their previous infection.^24^ One group suggested that the pandemic has created a “perfect storm in which nocebo effects may be flourishing,” including a flood of negative information from the media, and the fear and anxiety of negative expectations.^218^

The effect of vaccination on reducing the risk of long-COVID has been estimated at 15% or higher.^72,219,220^ Although some have hypothesized, in our view implausibly, that this reflects “autoimmune processing being ‘reset’ by vaccination . . . [or] any residual viral reservoir may be destroyed by antibody response,” one “cannot rule out the possibility of a change in reported symptoms after vaccination being due to a placebo effect [i.e., reversal of the nocebo effect].”^221^ One study directly supported this theory by finding the efficacy of vaccination in preventing symptoms typical of Long-COVID to be the same as or better in test-negative controls compared to test-positive Omicron cases.^70,222^ Often, vaccination allows some freedom from social restrictions (e.g., being allowed to attend classes and participate in social interactions), which may improve symptoms of anxiety and depression.^223^

## Mechanisms of functional disorders

### The BPCM for perception

**Figure 1.**
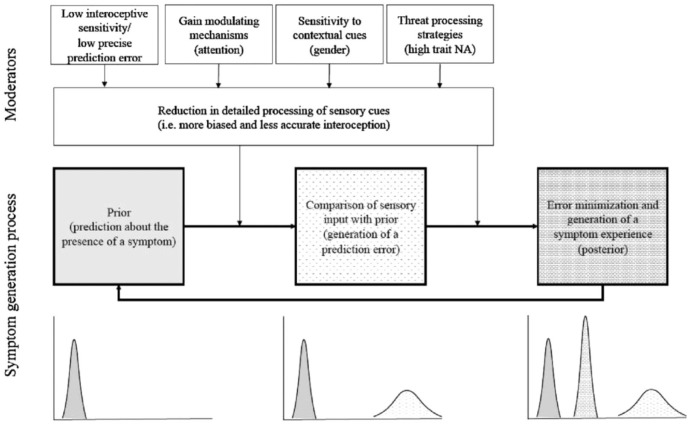
Bayesian predictive coding model of symptom perception. The prior (expectations based on previous symptom experience episodes) is compared with the afferent sensory input (observation) leading to a prediction error. To minimize error, a posterior inference (symptom experience) is generated that best matches the prior and prediction error. This posterior inference then becomes the prior input in a new symptom perception episode. Moderators can cause less precise/accurate afferent sensory input/processing, which leaves more room for the prior expectation to determine perception (see Figure 2). NA, negative effect. Figure (with modified legend) reproduced with permission from Elsevier (license 5507850839046), from Van den Bergh et al.^225^

**Figure 2.**
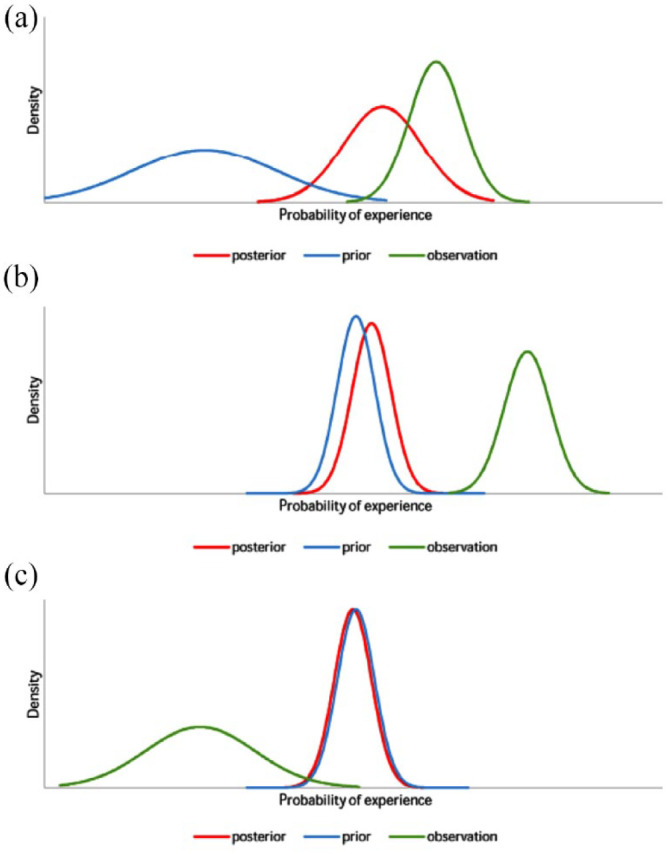
The Bayesian predictive coding model of inference by the brain. In panel (a) with a low precision prior, new information (observation) has a large impact on the formation of a posterior interpretation. In panel (b) with a high precision prior, new information (observation) has limited impact on the formation of a posterior interpretation. In panel (c) again with a high precision prior, low precision new information (observation) has little impact on the formation of a posterior interpretation (percept of a symptom). Figure (with modified legend) reproduced with permission from Elsevier (license 5507850839046), from Van den Bergh et al.^225^

Instead of passively awaiting sensory input, the brain is an active inferential machine. The brain hierarchically integrates sensory information (bottom-up input) and internal predictions about the expected information (top-down prior beliefs), each weighted according to their precision (mediated by top-down synaptic gain), to reach a posterior inference about what has happened (i.e., the percept).^160–162,213,221,224–230^ In Bayesian terms, this hierarchical process involves top-down prior predictions (represented by expectations), bottom-up likelihood (the Bayes Factor, represented by sensory input), and bottom-up prediction error (represented by the difference between the likelihood and prior, weighted by the precision of these two signals), to reach the posterior inference (represented by the percept) (see Figures 1 and 2).^160–162,213,221,224–230^ In this model, all experienced symptoms occur along a continuum of objectivity, more or less accurately representing what has happened to the body, and sometimes mistaking noise (normal bodily processes) for symptoms.^224,226,229,230^ In functional symptoms and syndromes, inaccurate inferences (so-called somatovisceral illusions or false perceptions) are made about the state of the body due to overly precise prior expectations overriding any bottom-up sensory data.^160,162,213,221,224–226,228–230^ In other words, the multi-network inferential *functioning* (i.e., functional connectivity) of the brain is (reversibly) abnormal, making “the brain’s best-guess [hypothesis] about the world [perception]” inaccurate, without structural pathological changes in either the body or the brain.^226^ Very importantly, the functional disturbance at lower levels of symptom perception is unconscious and beyond naïve dualistic mind–body models.^161,224,225,231^ At higher levels, the functional disturbance involves too much confidence in prior predictions of concern, leading to the attenuation of disconfirming evidence by negative cognitive reappraisal.^231^ This model can also explain placebo and nocebo effects that occur from abnormally precise prior expectations.^213,225–227^

### How Bayesian predictive coding can lead to perceptual illusions

Several factors modulate symptom perception in this model (see Figures 1 and 2). First, the type of sensory input. When the input is less intense, more systemic and widespread, with poor on/off boundaries (e.g., fatigue or malaise), or when there is interoceptive dysfunction with low signal to noise (e.g., during chronic stress with cytokine and stress-axis activation), this decreases precision of sensory input, leaving more room for prior expectations to determine perception.^224,225^ Conversely, when input is associated with strong cues (e.g., prior viral infection symptoms or panic attacks), these cues can later activate strong prior expectations that override incoming sensory noise.^221,224,225^ Second, the focus of attention. Attention can be thought of as modulating the balance of precision weights of sensory input and prior expectations. Body focus can lead to a self-fulfilling cycle, with bodily scrutiny (especially in the context of cues suggesting a possible health threat, or recent illness, injury, or emotional arousal) and confirmation bias (making relatively weak everyday interoceptive stimulation represented as stronger and more precise, especially when there is also negative affect) leading to updated priors that are excessively precise (i.e., reinforcing the learning of stronger illness beliefs), until the priors are precise enough to have noise represented as signal (i.e., as posterior percept).^213,221,225,229^ Third, genetic influences. For example, females appear to be more sensitive to contextual cues that influence prior expectations.^225^ Innate suggestibility has some genetic component and is increased under conditions of stress and trauma.^213^ Fourth, trait anxiety, especially in the face of anticipated threat and negative affect (with harm avoidance, catastrophizing, and fear). Chronic worry and stress can create a vicious circle, with elevated threat and salience detection (e.g., activating priors that predict threat, and that are categorically precise), and leading to active inference (e.g., activation of the autonomic nervous system, endocrine and immune systems, to produce low-precision input that conforms to the prior predictions) that reinforces the prior belief.^213,224–226,229^ Fifth, top-down prior beliefs and expectations. Self-fulfilling health-related expectations can lead to pathologically precise prior beliefs.^213^ This includes cultural expectations; for example, whiplash injury is rare in countries where the concept is not known, and a campaign to change expectations of consequences of minor injury led to a reduction in the population of chronic back pain.^221^ Worrisome ideas (about cause, significance, and prognosis of symptoms) and expectations about illness can originate from the social context, including from health scares in the media or from physicians, or illness in family or friends, and can lead to selective body-focused monitoring.^221,224,225,229^

All of these factors can lead to three incorrect inferences made during hierarchical Bayesian brain processing^213,221,224^: (1) “Autonomous emergence of a percept or belief that is held with undue certainty (precision) following top-down attentional modulation of synaptic gain.”^221^ (2) This percept “is falsely inferred to be a symptom to explain why its content was not predicted by the [higher level] source of attentional modulation.”^221^ (3) Active inference, where interoactions lead to sensory input that conforms to predictions and reinforces the precision of abnormal prior expectations.^213,224,227^ In the end, “symptoms unfold increasingly independent of actual physiological changes over the course of somatic symptom and related disorders . . . [with] symptom report decoupled from sensory input.”^230^ Cardinal features of these amplified symptoms include disproportionate findings of physical distress (e.g., exceptionally noxious and disruptive symptoms, and multiplicity of symptoms), negative cognitions (e.g., “unnecessarily negative expectations, unduly alarming suspicions, troubling interpretations, worrisome beliefs about their significance and cause,” including the conviction that an undiagnosed disease is present), health-related anxiety and disease fear, impairment of function (e.g., illness and sick-role behaviors, such as excessive use of medical care, information seeking, reassurance seeking, and avoidance of activity suspected of worsening symptoms), pervasiveness (e.g., preoccupied with symptoms that become part of self-identity), and dissatisfaction with medical care.^229^

This unifying theoretical account strengthens the case that Long-COVID is usually a functional syndrome that was predictable given the common pandemic responses described below.

## Implications for management of Long-COVID

### Reduce modifiable predisposing factors for functional syndromes

This would entail reducing health anxiety (including worry and catastrophizing), depression, fear (including anticipated threat and unduly negative expectations), negative media coverage (including by medical “experts”), social isolation, and physical inactivity. We have several suggestions to consider.

First, provide accurate information about risk to reduce anxiety and fear. The median infection fatality rate (IFR) from SARS-CoV-2 infection, prior to vaccines, was 0.034% for those aged 0–59 years, and 0.095% for those aged 0–69 years.^232^ The median IFR by age group was a median 0.0003% at 0–19 years, 0.002% at 20–29 years, 0.011% at 30–39 years, 0.035% at 40–49 years, 0.123% at 50–59 years, and 0.506% at 60–69 years.^232^ For those <70 years, this is 0.33%/0.095% = 3.5× lower than the *case* fatality rate in Canada in March 2021; correcting for this difference between case and infection outcome rates, the infection hospitalization and ICU admission rates for those <70 years in Canada were 3.0%/3.5 = 0.86% and 0.7%/3.5 = 0.2%, respectively, in May 2021^233^ (and with Omicron variants is now likely 3.5× lower).^234,235^ From a public health view, serious outcomes from SARS-CoV-2 are rare in these age groups, particularly in children. In adults aged >70 years living in the community, the median IFR before vaccines and Omicron variants was 2.2%, and focused protection, especially in those with multiple comorbidities, can be offered.^236^ Explaining that Long-COVID is usually not due to irreversible tissue damage, can be expected to be reversible, and is not much more common after COVID-19 infection than in non-infected controls, also can reduce fear and negative expectations. Second, lockdowns have led to increased anxiety, depression, social isolation, and physical inactivity, yet did not reduce COVID-19 rates or mortality in the population.^233,237–239^ Although physical activity decreased markedly during lockdown, physically active people were less likely, when infected, to report prolonged symptoms (OR 0.24; 95% CI 0.10, 0.55).^240^ Similarly, closing schools led to severe adverse effects on children’s mental health and learning, yet were not effective at reducing the COVID-19 burden.^233,237,241–251^ Not implementing lockdowns (including not closing schools), and explaining that they have a very negative cost–benefit ratio, is important.^233,241^ Third, negative media coverage must be replaced with accurate information. This requires repeated messaging by trusted leaders who explain the cost–benefit trade-offs of interventions and explain risk in the context of other risks we have always tolerated in order to have a democratic and free society.^233,238^ Fourth, ensuring that health leaders create surge capacity in hospitals for patients with and without COVID-19 during endemic respiratory viral seasons, instead of creating fear that hospitals are overwhelmed (and implementing cruel visitation policies), is also important.^233^ In Canada, hospitals have often been well above capacity in prepandemic years,^252–254^ and we suggest that it would be better to fix this problem instead of diverting attention by inducing fear and anxiety.^255,256^ Finally, masking signals to others (and reinforces) a fear of viruses including SARS-CoV-2, and this affective problem “is a contagious one: fear spreads among the public, leading to intensification of risk management.”^257^ The best evidence from before the pandemic,^258–260^ the community randomized trials during the pandemic,^261–264^ updated evidence after the pandemic,^265^ and observational school masking studies during the pandemic^266–268^ support that universal masking (especially) outside of hospitals is not effective in reducing transmission. One way to signal that it is time to move away from fear may be to abandon talk of community mask mandates.

### Reduce modifiable perpetuating factors for functional syndromes

This would entail reducing physical inactivity, social isolation, health anxiety (with unhelpful cognitions and fears), depression, social reinforcement, and contagion by (sometimes dramatic) exaggeration in conventional and social media, and late or missed diagnosis. We have several suggestions to consider.

First, the same suggestions discussed above to reduce predisposing factors will also reduce perpetuating factors. Second, de-escalating social reinforcement (and contagion). The focus from leaders, “experts,” social media influencers, and conventional media should be on providing accurate and therefore reassuring information about Long-COVID, without unduly exaggerated claims that perpetuate fear. Clear explanations (including by treating clinicians) should emphasize the functional nature of the condition (e.g., “a software not hardware problem” in perception), the lack of structural damage to the brain or organs, and the reversibility of the syndrome.^158,160^ Explanation should avoid problematic statements such as “all the tests were normal, so there is no disease,” “this is a psychiatric condition,” or “this is all in the mind”; these are not only inaccurate but also reduce trust and increase problematic cognitions.^138,158,160^ Third, at the individual level, therapy should be multidisciplinary (e.g., involving somatic rehabilitation clinics that existed prior to the pandemic), and focus on delivering and explaining the functional diagnosis, exercise and physical rehabilitation, and cognitive behavioral therapy.^136,138,158,160,162^ Follow-up is important to prevent the patient from feeling abandoned or ignored.^136,138,158^ Cognitive behavioral therapy aims to change maladaptive thoughts (e.g., symptom focusing, believing symptoms are a sign of damage, catastrophizing) and behaviors (e.g., avoidance of social interaction or physical activity).^127^ Improving sleep hygiene, and treating comorbid anxiety and depression can also be helpful.^136,140,162^

### Some important clarifications

First, the SARS-CoV-2 pandemic response did not follow previous pandemic plans nor the emergency management process, leading to the predisposing, precipitating, and perpetuating factors described above. What a better response would look like, led by emergency management experts following the emergency management process, is beyond the scope of this review, and is described elsewhere.^233,256,269–271^ Second, in functional syndromes, the symptoms and disability are real and genuinely experienced, and not feigned or faked. Third, clinicians must take the symptoms and disability seriously, and explain clearly that there is a diagnosis, this diagnosis is common, the mechanism is functional, and therefore the condition is potentially reversible and treatment can help. Fourth, we acknowledge that some Long-COVID cases will be due to pathological structural disease, and some investigations will be important and necessary to rule these out in individual cases according to physician judgment. This may be particularly true for the most severe cases of acute COVID-19, especially those with post-intensive-care syndrome. Indeed, in a population-based cohort study in Ontario, Canada, the risk of incident cardiovascular, neurological, and mental health conditions and rheumatoid arthritis more than 30 days after hospitalization for COVID-19 was comparable with other acute infectious illnesses (e.g., influenza and sepsis), suggesting these disorders “may be related to the severity of infectious illness necessitating hospitalization, rather than being direct consequences of infection with SARS-CoV-2.”^272^ Fifth, although having COVID-19 viral illness can be a precipitating factor for functional syndromes, we believe this is far less common than people have been led to believe, and the more common precipitating factors may be psychosocial trauma and belief in the threat of Long-COVID (along with the dramatic public health and media response, creating stress and fear).

Sixth, our hypothesis avoids the accusation of “medical gaslighting,” by attempting to maintain epistemic humility and avoid ontological politics.^11,57^ Specifically, we explicitly suggest not dismissing patient descriptions of their symptoms; accepting patient reports as describing a genuine illness associated with significant suffering; providing supportive, empathetic, and timely medical diagnosis; and offering timely multidisciplinary treatment including CBT and graded exercise. We also argued that symptoms cannot be dismissed as simply the “product of anxiety” (e.g., don’t turn a predisposing factor into the “cause”) or “a mental problem” (e.g., all symptom perception depends on the same brain mechanisms). In addition, we offered a physiological explanation for the syndrome, that is, the brain’s Bayesian perceptual processing. Accordingly, it would be a mistake to think we suggest that Long-COVID is “only a psychological problem”; this would be a belief that mistakenly perpetuates mind–body dualism, and misunderstands the mechanisms of brain functioning and perception.

## Limitations

First, this was not a systematic review, and we may have missed important studies contrary to our hypothesis. We refer to many systematic reviews to support our argument from analogy. These reviews consistently found that, although there are many hypothesized mechanisms, the symptoms of Long-COVID remained largely “medically unexplained” (Table 2). Second, there may be other pathophysiologic mechanisms for Long-COVID. We refer to hypotheses of organ damage, viral persistence, autoimmunity, and neuroinflammation, and argued that findings are inconsistent at best (Table 2) and that biomarkers of inflammation sometimes found in Long-COVID are nonspecific and of unclear cause–effect relationship.

This is not the view taken by many authors; however, those authors did not consider the mechanism we suggest, as demonstrated in a systematic review of functional neurologic symptoms in Long-COVID.^273^ For example, CFS (also occurring in Long-COVID) has been thought due to mitochondrial pathology or abnormal inflammation, and CBT and graded exercise therapy have been suggested to worsen outcomes.^86,274–276^ Yet, recent systematic reviews of CFS found that “it is difficult to establish the role of mitochondria in the pathomechanisms of ME/CFS/SEID due to inconsistencies across studies,”^277^ that “there are few consistent [immunological] findings and there is almost a complete lack of longitudinal studies,”^278^ that the quality of studies for or against CBT or graded exercise therapy was often low,^276^ and that CBT, graded exercise therapy, and pacing are effective therapies.^279–282^ The finding in large cohort studies that “patients with mild Covid-19 are at risk for a small number of health outcomes, most of which are resolved within a year from diagnosis,”^283^ and that workers’ compensation claims have “fallen sharply over time” with “approximately 18% of claimants with Long Covid . . . unable to return to work for more than one year,”^284^ seem contrary to views that Long-COVID is a long-term disease that does not respond to therapy.^285^ The only randomized controlled trial (RCT) that we are aware of studying CBT for severe fatigue in Long-COVID, found that CBT was effective in reducing fatigue, with positive effect sustained at 6-month follow-up.^286^ Similarly, the only RCT of exercise for long-COVID that we are aware of found that a supervised exercise intervention at low and moderate intensity was a “more effective, safe, and well-tolerated intervention in post-COVID-19 conditions” than usual care.^287^

Third, our hypothesis has not been tested. Long-COVID studies have rarely considered functional diagnoses, nor systematically looked for positive features of functional disorders (e.g., inconsistency over time, modulation by attention, distractibility) including FCD.^273^ Whether long-COVID can be prevented or treated in the ways we suggested requires future research that we believe is extremely important. Fourth, whether long-COVID is one or many different conditions is unclear, and whether all or even most are explained by our hypothesis can only be determined by future studies. We make this hypothesis so that it can be tested in future studies, thus aiming for scientific progress in explaining and treating Long-COVID.

## Conclusion

We used an argument by analogy, reviewing what is known about Long-COVID, pandemic response effects on mental and physical health, functional syndromes including mass sociogenic illness, and the unifying mechanism among these (with common predisposing, precipitating, and perpetuating factors, Table 4), to offer an alternative perspective—that the majority of Long-COVID is a functional disorder. We discussed the implication that current pandemic response strategies have been causing predisposing, precipitating, and perpetuating factors for Long-COVID. Perhaps a better term for the syndrome than “Long-COVID” or “Long-Pandemic” would be “Pandemic-Response Syndrome,” to better reflect the etiologic factors we propose, and to serve as a lesson learned, not to be repeated in the future. Ultimately, we aimed to help the many people suffering from Pandemic-Response Syndrome.

**Table 4.** Predisposing, precipitating, and perpetuating factors that are remarkably similar among Long-COVID and functional disorders, and how the Bayesian predictive coding model for perception can explain these factors.

Disorder	Predisposing	Precipitating	Perpetuating
Long-COVID	-Female gender (post-pubertal) -Poor prior physical or mental health, including preexisting anxiety, worry about COVID-19, depression, or functional syndromes -Feeling of loneliness -Asthma or other causes for chronic dyspnea	-More severe acute-COVID-19: more symptoms, hospitalization, intensive care admission, or dyspnea -Belief in having been infected with COVID-19	-Symptoms in household members
Somatic symptom disorders, syndromes, and functional neurological disorders	-Female gender (post-pubertal) -Anxiety and fear -Depression -Traumatic experiences, including stress -Autonomic hyper-arousal (e.g., panic attacks) -Childhood adversity	-Acute illness or trauma (e.g., traumatic brain injury or concussion, medical event, viral illness) -Psychosocial adversity/trauma -Autonomic hyper-arousal (e.g., panic attacks)	-Poor physical activity -Poor social activity -Poor sleep hygiene -Missed/late diagnosis -Unhelpful cognitions including fear of organ damage and a missed physical cause -Social reinforcement, including by media
Mass sociogenic illness	-Female gender -Anxiety -Depression -Psychological distress and fear -Unhealthy behaviors (e.g., unhealthy eating, lack of exercise, disordered sleep, lack of socialization) -Popular health fears -Negative expectations (nocebo effects)	-Environmental event with a dramatic emergency response and with fear of an unpredictable threat -Traumatic experience e.g., war	-Social contagion: proximity to others who are ill, the excitement of the negative media coverage (including social media), labeling the illness -Having many medical investigations -Negative expectations (nocebo effects)
Within the Bayesian predictive coding model for perception	-Type of sensory input: less intense, more systemic and widespread, or interoceptive dysfunction due to chronic stress -Body-focused attention and scrutiny -Genetic: female gender, suggestibility -Anxiety and negative affect, e.g., chronic worry and stress -Active inference, e.g., activation of autonomic, endocrine, and immune systems -Prior negative beliefs and expectations	-Strong cues (e.g., viral infection symptoms, panic attacks) create a vicious circle that results in abnormally precise prior expectations	-Self-fulfilling cycles that reinforce prior expectations, e.g., ongoing body-focused attention, health anxiety and fear, cultural beliefs, and worrisome ideas about illness.

For more details and many references, please see the text.
